# Physical Activity Regulates TNFα and IL-6 Expression to Counteract Inflammation in Cystic Fibrosis Patients

**DOI:** 10.3390/ijerph18094691

**Published:** 2021-04-28

**Authors:** Ersilia Nigro, Rita Polito, Ausilia Elce, Giuseppe Signoriello, Paola Iacotucci, Vincenzo Carnovale, Monica Gelzo, Federica Zarrilli, Giuseppe Castaldo, Aurora Daniele

**Affiliations:** 1 Dipartimento di Scienze e Tecnologie Ambientali, Biologiche, Farmaceutiche, Università della Campania “Luigi Vanvitelli”, 81100 Caserta, Italy; nigro@ceinge.unina.it (E.N.); polito@ceinge.unina.it (R.P.); 2 CEINGE-Biotecnologie Avanzate Scarl, Via G. Salvatore 486, 80145 Napoli, Italy; ausilia.elce@unipegaso.it (A.E.); monica.gelzo@unina.it (M.G.); federica.zarrilli@unina.it (F.Z.); giuseppe.castaldo@unina.it (G.C.); 3 Dipartimento di Sanità Pubblica, Università degli Studi di Napoli “Federico II”, 80131 Napoli, Italy; 4 Dipartimento di Scienze Umanistiche, Università Telematica Pegaso, 80132 Napoli, Italy; 5 Dipartimento di Salute Mentale e Fisica e Medicina Preventiva dell’Università degli Studi della Campania “Luigi Vanvitelli”, 80138 Naples, Italy; giuseppe.signoriello@unicampania.it; 6 Centro Regionale Fibrosi Cistica Adulti, Dipartimento di Scienze Mediche Traslazionali, Università degli Studi di Napoli Federico II, 80131 Napoli, Italy; paola.iacotucci@unina.it (P.I.); vincenzo.carnovale@unina.it (V.C.); 7 Dipartimento di Medicina Molecolare e Biotecnologie Mediche, Università degli Studi di Napoli Federico II, 80131 Napoli, Italy

**Keywords:** cystic fibrosis, physical activity, TNFα, IL6, inflammation

## Abstract

Background: Cystic fibrosis (CF) is one of the most common inherited diseases. It is characterised by a severe decline in pulmonary function associated with metabolic perturbations and an increased production of inflammatory cytokines. The key role of physical activity (PA) in improving the health status of CF patients and reducing lung function decline has recently been demonstrated. This study evaluated interleukin-6 (IL-6) and tumour necrosis factor α (TNFα) expression in two subgroups of CF patients classified based on PA. Methods: We selected 85 CF patients; half of them regularly undertook supervised PA in the three years leading up to the study and half of them were not physically active. Patients were analysed for serum IL-6 and TNFα levels using enzyme-linked immunosorbent assays. Results: We found that the expression levels of IL-6 and TNFα differed in terms of their regulation by PA. In particular, TNFα levels negatively correlated with FEV1% decrease/year and FEV1% decrease (*p* = 0.023 and *p* = 0.02, respectively), and positively correlated with serum fasting glucose (*p* = 0.019) in PA CF patients. In contrast, in the NPA subgroup, TNFα levels were positively correlated with IL-6 (*p* = 0.001) and negatively correlated with adiponectin (*p* = 0.000). In addition, multiple logistic regression analysis confirmed that PA is an independent modulator of the inflammatory state. Conclusions: PA modulates inflammatory processes in CF patients by regulating the secretion of pro-inflammatory cytokines and thus ameliorating lung function. Our data show that PA is a useful complementary strategy in the management of CF and that TNFα may be a marker of these effects of PA.

## 1. Introduction

Cystic fibrosis (CF) is the most common autosomal recessive disorder in Caucasian populations caused by mutations in the cystic fibrosis transmembrane conductance regulator (*CFTR)* gene [1]. 

*CFTR* gene, located on the long arm of chromosome 7, encodes a 1480 residue transmembrane glycoprotein. To date, approximately 2000 different mutations have been described, although the most common disease-causing mutation in CF patients is p.Phe508del [2]. There are environmental risk factors associated with aggravation of lung and muscle functions. Physical inactivity and additional factors (e.g., inflammation, metabolic abnormalities) are probably the major contributors to muscle and lung abnormalities in CF patients [3]. 

In CF, inflammation plays a critical role in lung pathology and disease progression; this makes the disease an interesting area of research with broad therapeutic relevance [4,5,6]. Indeed, in the airways of CF patients, the epithelial cells become deficient in transmembrane conductance regulator (CFTR) through abnormal processes; as such, the transcription of inflammatory molecules is activated [7]. The dysregulation of epithelial cells and innate immune function in the lungs results in increased, albeit ineffective, inflammation [8]. CF patients show progressive deterioration of lung function associated with the increased production of pro-inflammatory cytokines such as tumour necrosis factor α (TNFα), interleukin-6 (IL-6), and IL-8 in serum and in saliva [9,10]. In this scenario, physical activity (PA) plays a pivotal role in the treatment of chronic diseases characterised by increased inflammation status, including metabolic pathologies [11]. Indeed, moderate-intensity exercise is considered to enhance immune function and to be useful in preventing acute upper respiratory infections and similar conditions [11]. In particular, exercise training improves symptom burden, exercise capacity, and peripheral muscle function at all stages of COPD [12]. Additionally, the anti-inflammatory effects of exercise training are well established in healthy subjects and patients with cancers and/or cardiovascular diseases [13]. Physical activity suppresses systemic inflammation via local muscle release of myokines that are integral part of overall organ crosstalk [14]. The most expressed myokine is interleukin-6 (IL-6), which is involved in the metabolic and immunological response to exercise [15]. 

Likewise, a not physically active lifestyle negatively impacts general health status and increases susceptibility to infections [16]. It was recently demonstrated that long-term PA offers significant beneficial effects in terms of decline in percentage of predicted forced expiratory volume (FEV1%), lipid and glucose metabolism, and inflammation in CF patients [17,18]. Furthermore, it was demonstrated that adipose tissue is involved in the changes stimulated by PA, as adiponectin levels significantly decrease in CF patients after extended and regular PA [18]. The literature also indicates that regular and structured PA mediates beneficial effects through key biological processes involving the cytokine crosstalk between the skeletal muscle, the immune system, and adipose tissue [19,20]. Indeed, the anti-inflammatory effects of exercise include suppression of the recruitment and activation of neutrophils and monocytes/macrophages, principal sources of inflammatory mediators such as cytokines [21]. These anti-inflammatory effects seem to be effective in the prevention of chronic inflammation in various diseases. 

It is worth considering that the inflammatory milieu is important in various pathophysiological lung conditions, although there has been no knowledge of shared blood inflammatory biomarkers until now. Candidate biomarkers may potentially reveal the effects of exercise from a pathological point of view, thus becoming early prognostic markers.

It is important to test whether lung function parameters can be improved by regular and prolonged PA and which molecular mediators are implicated. In this context, the aim of this ancillary study was to evaluate the anti-inflammatory effects of exercise. As such, we examined the expression of TNFα and IL-6 in two subgroups of CF patients classified on the basis of lifestyle; the first group comprised physically active patients (termed PA) and the second group comprised not physically active patients (termed NPA). We also measured correlations between biochemical and clinical parameters in the two subgroups. 

## 2. Materials and Methods

The study population consisted of 85 patients diagnosed with CF [22] from the regional CF care centre of Campania, Southern Italy. Inclusion criteria were as follows: aged over 18 years, pathological SCL (chloride > 60 mEq/L), and two class I–II CFTR mutations. Exclusion criteria were as follows: presence of acute phase of respiratory infection. Of the recruited patients, 42 regularly undertook PA in the last three years (i.e., the PA group), while 43 age-matched CF patients had not undertaken regular PA (i.e., the NPA group) in the last three years. Each patient performing physical exercise (pure respiratory muscle training excluded) was helped to individually select their exercise program under the supervision of a physician trained in CF and a physiotherapist, based on the severity of their disease. All patients in the PA group undertook a combination of aerobic and anaerobic training. The study team was responsible for monitoring the compliance of the patients and preventing complications related to physical activity. Supplementary Table S1 reports details about the physical activity program followed by the PA CF group (weekly frequency, minutes/session, and minutes/week). The best FEV1 values were recorded, expressed as percentage of predicted value for age based on standardised reference equations for spirometry [23]. Using blood specimens collected at the time of sampling for diagnostic purposes, IL-6 and TNFα serum concentrations were evaluated using a commercially available enzyme-linked immunosorbent assay (ELISA) kit (MAX Deluxe Set Human IL-6, BioLegend, San Diego, CA, USA). Homeostasis model assessment of insulin resistance (HOMA-IR) was assessed. The research protocol was approved by the Ethics Committee of the School of Medicine, Università di Napoli “Federico II” (Prot.n. 78/21) and was conducted in accordance with the principles of the Helsinki II Declaration. Written informed consent was obtained from all participants. All data are expressed as the mean ± SD. The Kolmogorov–Smirnov test was used to test the normality of distributions. Student’s *t*-test or the Mann–Whitney U test for independent samples were used to compare parametric and nonparametric numerical data, respectively. Frequencies of categorical variables were compared using the Chi-square test. IL-6 and TNFα serum concentrations were correlated based on Pearson or Spearman’s rho tests, according to the distributions of the data. A post hoc sample size of 43 in each group had 80% power to detect a difference in means of 0.920 (the difference between a Group 1 mean, m1, of 13,800 and a Group 2 mean, m2, of 12,880) assuming that the common standard deviation was 1500, using a two-group *t*-test with a 0.050 two-sided significance level. Odds ratios (ORs) with 95% confidence intervals (CIs) were estimated using a logistic regression model with the significant variables of the univariate model as covariates. A *p* < 0.05 was considered statistically significant. Statistical analyses were conducted using the Statistical Package for Social Sciences software for Windows (SPSS, Inc, Chicago, IL, USA).

## 3. Results

Table 1 shows the anthropometric, biochemical, and disease-specific parameters of the two CF patient subgroups: those who regularly undertook PA over the last three years and not physically active CF patients. 

Adiponectin and leptin levels and FEV1% values have previously been evaluated in CF patients [18]; active CF patients had lower FEV1% decrease values than the not physically active CF patients. In addition, adiponectin levels were statistically lower in PA than in NPA patients (12.9 ± 2.0 versus 13.8 ± 2.2, *p* = 0.049). We evaluated TNFα serum concentrations in 31 CF patients who undertook PA compared to 33 not physically active CF patients, and compared the IL-6 serum concentrations in 42 CF patients that undertook PA compared to 43 not physically active CF patients. The IL-6 serum concentrations were higher in the PA CF patients than in their NPA counterparts (50.8 ± 17.4 versus 44.7 ± 12.4). The TNFα serum levels were significantly higher in not physically active CF patients than in active patients (12.6 ± 5.1 versus 10.6 ± 1.8, *p* = 0.043). The HOMA-IR index was significantly higher in NPA patients than in active patients (1.46 ± 1.13 versus 2.24 ± 2.05, *p* = 0.043). The PA patient subgroup had a reduced rate of hospitalisation and showed an attenuated (not significant) lung function decline as indicated by both FEV parameters (see Table 1).

Table 2 presents the results of the correlation analysis: TNFα levels were negatively correlated with FEV1% decrease parameters (*p* = 0.023 and *p* = 0.02, respectively) and positively correlated with serum fasting glucose (*p* = 0.019) in PA CF patients. In contrast, in the NPA subgroup, TNFα levels were positively correlated with IL-6 (*p* = 0.001) and negatively correlated with adiponectin (*p* = 0.000). In addition, in the same subgroup, IL-6 was negatively correlated with leptin (*p* = 0.008). 

We performed a multiple logistic regression analysis which confirmed that PA is an independent modulator of the inflammatory state in CF; indeed, our findings confirm that TNFα, HOMA-IR, and RD are variables significantly associated with PA. Gender, comorbidities, and age were not confounding factors (Table 3).

## 4. Discussion

Cystic fibrosis is a genetic disease characterised by a progressive decline of pulmonary function, which seriously affects the quality of life of patients. The lung damage is caused by an exaggerated inflammatory process in which TNFα plays a central role [14]. 

In this context, this ancillary study aimed to investigate the beneficial effects of a long-term, regular physical activity regime in CF patients by examining different systemic parameters and focusing on TNFα and IL-6 levels. Multiple reports have demonstrated that physical exercise contributes improvement of different aspects of human life by ameliorating the release of inflammatory mediators [24]. One of the key mechanisms by which PA exerts favourable health effects is via the stimulation of an interorgan crosstalk (skeletal muscle, adipose tissue, and immune system). The mechanisms through which PA induces increased insulin sensitivity as well as ameliorating inflammation are not yet fully clarified, but are probably mediated by soluble muscle endocrine mediators such as IL6 [25]. Accordingly, in this study, we found that in the subgroup of CF patients that undertook PA for three years, there was a statistically significant decrease of glycaemia, HOMA-IR index, and adiponectin and TNFα levels. In addition, it was possible to observe a global health status improvement of PA CF patients compared to the NPA patients (see Table 1). Indeed, although not significant, the PA patient subgroup had a reduced rate of hospitalisation. Regression statistical analysis confirmed that PA is an independent modulator of inflammatory state in CF, as indicated by the reduction of TNFα. However, the limited number of CF patients analysed is a limitation of the study.

TNFα plays a central role in the inflammatory status of CF patients; it is harmful in many organs and tissues, including the lungs. IL-6 is a multifunctional cytokine produced by many kinds of immune cells [26,27]. In addition, recent evidence has demonstrated that skeletal muscles release significant amounts of IL-6 in response to contraction [28], which contributes to mass gain [29]. 

Among its other benefits, PA can increase adiponectin levels in healthy individuals [30]. A previous study investigated the effects of PA on adiponectin and leptin levels in CF patients, demonstrating that adipose tissue is involved in the inflammatory process underlying CF and is related to a poorer lung phenotype [18]. In addition, a lower exercise capacity has been associated with a higher mortality rate, steeper decline in pulmonary function, and higher inflammation [31,32]. The present study further examined the relationship between inflammation, physical activity, and clinical phenotype in CF, demonstrating that TNFα levels are statistically lower in active CF patients and negatively correlate with adiponectin while being positively associated with IL-6. The increase in IL-6 following PA has been described as an anti-inflammatory mechanism and a regulator of protein synthesis and body composition [33,34]. Furthermore, IL-6 has been described as a major contributor to the anti-inflammatory effects of exercise in both healthy individuals and patients affected by metabolic disorders undertaking regular PA [33,35]. However, reports in the literature describe IL-6 as both a pro-inflammatory (when produced by macrophages) and an anti-inflammatory (when produced by muscle) molecule depending on the environment [29].

PA counteracts inflammation through several molecular mechanisms, including the downregulation of TNFα levels [36]. In line with this evidence, the data analysed in this study show that PA induced a decrease of TNFα in CF patients. The observed changes in systemic cytokines, which are likely initially determined by the overall activation status of the immune cells, could in turn intensify their recruitment and stimulation (especially of neutrophils) at both the lung and systemic levels of CF patients that do not undertake PA [37,38,39]. Indeed, it has been reported that TNFα sustains the recruitment and activation of both neutrophils and macrophages, contributing to establishment and exacerbation of the chronic inflammatory state in different organs [27,40]; in contrast, a decrease in the levels of this cytokine (which we observed in CF patients regularly undertaking PA) may substantially contribute to reduction of the recruitment of immune cells and thus the inflammatory process [27,40]. We hypothesised that in the complex inflammatory milieu typical of CF, PA contributes to regulation of the immune system by acting on the lungs as well as on different organs and tissues; in particular, our data focused on adipose tissue, which seems to have a key role, as suggested by the amelioration of the lipid profile and adipokine secretion. Thus, attenuation of the systemic and multi-organ inflammatory processes by PA in CF patients results in a general improvement of metabolic and respiratory health.

Interestingly, reduced inflammation due to PA results in an improvement of lung function, as demonstrated by the correlation between TNFα and FEV1% decrease in active CF patients. In contrast, the direct correlation between TNFα and IL-6 and the negative association between leptin and adiponectin in the NPA group suggests that the inflammatory status of these latter patients was ongoing and perpetuated by myokines and adipokines. In addition, the inverse correlation between TNFα and adipokines observed in NPA subgroup suggests that the interplay between adipokines and myokines in this patient subgroup adheres to the canonical mechanism of regulation occurring in the nonactive healthy population. 

## 5. Conclusions

In conclusion, although a causal relationship between attenuated FEV1% decline and decreased adiponectin and TNFα levels has not been clearly proven, this study demonstrated that PA may represent a useful complementary strategy in the management of CF patients and that these cytokines are valuable as blood markers of inflammation. Furthermore, serum TNFα may be used as marker to trace the beneficial effects of PA on patient health. PA contributed to amelioration of inflammation and respiratory function of patients. Further research is required to deeply investigate how these cytokines modulate inflammation and to test the efficacy of PA as a useful nonpharmacological coadjutant in classical CF patient treatment. Improvements in quality of life achieved by implementing not only exercise but also favourable lifestyle factors are anticipated to become more actively promoted for CF patient management in the future.

## Figures and Tables

**Table 1:** Clinical, anthropometric, and biochemical values in CF patients grouped on the basis of physical activity (mean values and standard deviation or number and frequency).

	PA CFGroup (*n* = 42)	NPA CFGroup (*n* = 43)	*p* Value
Age (years)	29.8 (8.4)	31.1 (9.1)	n.s.
Sex male/female	20/22	22/21	n.s. **
FEV1% decrease/year	0.60 (3.91)	1.65 (4.76)	n.s.
FEV1% decrease (%)	0.91 (4.80)	2.10 (6.79)	n.s.
Colonisation (*n*, %)			
by *P. aeruginosa*	28 (66.7)	28 (65.1)	n.s. **
by *S. maltophilia*	2 (4.8)	2 (4.7)	n.s. **
by *B. cepacia*	2 (4.8)	1 (2.3)	n.s. **
Hospitalisations (*n*, %)	4 (10.3)	11 (25.6)	n.s. **
CF-PI (*n*, %)	33 (78.6)	32 (74.4)	n.s. **
CF-LD (*n*, %)	7 (18.4)	5 (13.2)	n.s. **
CF-RD (*n*, %)	18 (42.9)	10 (23.3)	n.s. **
Body mass index (kg/m^2^)	22.5 (3.2)	22.1 (4.0)	n.s.
Serum fasting glucose (mg/dL)	86.3 (13.7)	91.9 (12.6)	n.s.
Insulinemia (mg/dL)	6.92 (5.82)	10.03 (9.77)	n.s. *
HOMA-IR	1.46 (1.13)	2.24 (2.05)	0.043 *
Adiponectin (µg/mL)	12.9 (2.0)	13.8 (2.2)	0.049
Leptin (ng/mL)	10.6 (11.8)	7.7 (8.1)	n.s. *
C-reactive protein (mg/L)	3.07 (3.79)	5.04 (6.26)	n.s. *
TNFα (pg/mL)	10.6 (1.8)	12.6 (5.1)	0.043
IL-6 (pg/mL)	50.8 (17.4)	44.7 (12.4)	n.s.

*p* values determined using *t*-test, * Mann–Whitney U test or ** Chi-square test; n.s.: not significant. PA: physical activity; NPA: no physical activity; PI: pancreatic insufficiency; LD: liver disease; RD: related diabetes; HOMA-IR: homeostasis model assessment of insulin resistance.

**Table 2:** IL-6 and TNFα correlations in CF patients grouped on the basis of physical activity.

	PA CF Group (*n* = 31)	NPA CF Group (*n* = 33)
TNFα		
IL-6		+0.563 (*0.001*)
Adiponectin		−0.594 (<*0.0001*)
FEV1% decrease/year	−0.408 (*0.023*)	
FEV1% decrease (%)	−0.417 (*0.020*)	
Serum fasting glucose	+0.420 (*0.019*) *	
IL-6		
Leptin		−0.460 *(0.008)* *

Coefficients and *p* values (italic number) determined by Pearson correlation or * Spearman’s rho correlation. PA: physical activity; NPA: no physical activity.

**Table 3:** Multiple logistic regression analysis of associations with physical activity.

	OR	95% CI per OR		*p* Value
		Lower	Higher	
Sex (M vs. F)	7.60	0.49	117.24	0.15
Age	0.97	0.85	1.12	0.70
Adiponectin	0.74	0.46	1.21	0.24
Leptin	1.07	0.96	1.18	0.24
IL6	1.03	0.94	1.14	0.49
TNFα	0.59	0.36	0.98	0.04
BMI (kg/m^2^)	1.32	0.81	2.14	0.26
LD (yes vs. no)	0.01	0.00	2.67	0.11
RD (yes vs. no)	50.87	1.87	1382.79	0.02
HOMA-IR	0.50	0.25	0.98	0.05
Glycaemia mg/dL	0.96	0.87	1.05	0.38

LD: liver disease; RD: related diabetes.

## Supplementary Materials

The following are available online at https://www.mdpi.com/article/10.3390/ijerph18094691/s1, Table S1. Physical activity program in PA CF group (mean values and standard deviation).
