# Supplementary material for: Physical Activity Regulates TNFα and IL-6 Expression to Counteract Inflammation in Cystic Fibrosis Patients
Source: Int J Environ Res Public Health. 2021 Apr 28;18(9):4691. doi: 10.3390/ijerph18094691 (PMC8125516; doi:10.3390/ijerph18094691)
Supplement: Supplementary file 1 [file ijerph-18-04691-s001.zip › ijerph-1182601-supplementary.pdf]

Table S1. Physical activity program in PA CF group (mean values and standard deviation).

	**PA CF group (n = 42)**
Weekly frequency (days)	3.43 (1.33)
Minutes/session	67 (34)
Minutes/week	221 (148)

PA: physical activity
